# Dauer Formation in *C. elegans* Is Modulated through AWC and ASI-Dependent Chemosensation

**DOI:** 10.1523/ENEURO.0473-20.2021

**Published:** 2021-04-08

**Authors:** Pratima Pandey, Umer S. Bhat, Anuradha Singh, Aiswarya Joy, Varun Birari, Nagesh Y. Kadam, Kavita Babu

**Affiliations:** 1 Department of Biological Sciences, Indian Institute of Science Education and Research (IISER) Mohali, Manauli, Punjab 140306, India; 2 Centre for Neuroscience, Indian Institute of Science, Bangalore, Karnataka 560012, India

**Keywords:** ASI neuron, *C. elegans*, chemotaxis, dauer, EXP-1, STR-2

## Abstract

The perception of our surrounding environment is an amalgamation of stimuli detected by sensory neurons. In *Caenorhabditis elegans*, olfaction is an essential behavior that determines various behavioral functions such as locomotion, feeding and development. Sensory olfactory cues also initiate downstream neuroendocrine signaling that controls aging, learning, development and reproduction. Innate sensory preferences toward odors (food, pathogens) and reproductive pheromones are modulated by 11 pairs of amphid chemosensory neurons in the head region of *C. elegans*. Amongst these sensory neurons, the ASI neuron has neuroendocrine functions and secretes neuropeptides, insulin-like peptide (DAF-28) and the TGF-β protein, DAF-7. Its expression levels are modulated by the presence of food (increased levels) and population density (decreased levels). A recent study has shown that EXP-1, an excitatory GABA receptor regulates DAF-7/TGF-β levels and participates in DAF-7/TGF-β-mediated behaviors such as aggregation and bordering. Here, we show that *exp-1* mutants show defective responses toward AWC-sensed attractive odors in a non-autonomous manner through ASI neurons. Our dauer experiments reveal that in *daf-7* mutants, ASI expressed EXP-1 and STR-2 (a G-protein-coupled receptor; GPCR) that partially maintained reproductive growth of animals. Further, studies suggest that neuronal connections between ASI and AWC neurons are allowed at least partially through ASI secreted DAF-7 or through alternate TGF- β pathway/s regulated by EXP-1 and STR-2. Together, our behavioral, genetic and imaging experiments propose that EXP-1 and STR-2 integrate food cues and allow the animals to display DAF-7/TGF-β neuroendocrine dependent or independent behavioral responses contributing to chemosensensory and developmental plasticity.

## Significance Statement

This work sheds light on a possible developmental and postdevelopmental function for the excitatory GABA receptor, EXP-1. We show that mutants of *exp-1* are defective in their response toward AWC-sensed odors. Our genetic, behavioral and expression studies reveal that EXP-1 functions in the ASI neuron to modulate chemosensation and to regulate the behavioral switch between dauer and the reproductive state. EXP-1 has been shown to function in a DAF-7/TGF-β-dependent manner. However, in the absence of DAF-7/TGF-β, EXP-1, and a G-protein-coupled receptor (GPCR), STR-2 integrate sensory information to maintain the reproductive state of the animal through an ASI-dependent alternate pathway.

## Introduction

*Caenorhabditis elegans* inhabit a continually changing environment and thus require very efficient sensory systems to perceive their surroundings for better survival. These animals largely rely on four major sensory systems: taste, touch, temperature and smell (Bargmann, 1998). *C. elegans* have highly evolved chemosensory abilities as this allows them to survive in unpredictable natural conditions to locate food and sense danger. As soon as the animal hatches into the first instar larval stage, its chemosensory abilities are fully functional and decides its future larval development (Golden and Riddle, 1984a). Sustainable food and growth conditions promote the reproductive cycle, whereas food scarcity and increased pheromone levels because of overcrowding promote entry into the dauer stage (Cassada and Russell, 1975; Golden and Riddle, 1984a,b).

Chemosensory signals are perceived through gustatory (water-soluble cues) or olfactory (volatile cues) amphid neurons (Bargmann et al., 1993). Olfactory cues are recognized from greater distances when compared with the gustatory cues (Bargmann and Mori, 1997). The AWC and AWA neurons in *C. elegans* detect volatile attractants whereas the AWB neuron is responsible for sensing aversive odors (Bargmann and Horvitz, 1991). Sensory neurons are in direct contact with the environment to perceive external cues, initiate neuroendocrine signaling that modulate vital developmental decisions and behaviors (Vowels and Thomas, 1994; Coburn and Bargmann, 1996; Daniels et al., 2000). To sense the environment very precisely with limited neurons, chemosensory neurons in *C. elegans* have evolved to express multiple receptors in contrast to mammalian neurons where each neuron harbor a single set of receptors (for review, see Hart and Chao, 2010). Spatial and temporal expression of many chemoreceptors is regulated by the DAF-7/TGF-β pathway (Zwaal et al., 1997; Birnby et al., 2000; Daniels et al., 2000; Sengupta, 2007). DAF-7/TGF-β is secreted from the ASI chemosensory neurons (amphid neurons) in response to environmental signals such as pheromones, pathogens, temperature and food. Defects in DAF-7/TGF-β signaling result in phenotypes such as constitutive dauer formation, increased life-span, aggregation and bordering (Thomas et al., 1993; Peckol et al., 2001; Cheung et al., 2004; Shaw et al., 2007). The levels of DAF-7/TGF-β were shown to be regulated by a GABA receptor, EXP-1 that also participates in aggregation and bordering behaviors seen in *daf-7* mutants (Bendesky et al., 2012).

The EXP-1 gene is an excitatory GABA receptor that is expressed in enteric muscles of the intestine, where it mediates muscle contractions in response to GABA signaling from motor neurons (Beg and Jorgensen, 2003). EXP-1 is also expressed in five sets of head neurons (SABD, RID, ADE, and two unidentified neurons; Beg and Jorgensen, 2003). It has been previously reported that GABA signaling in the accessory olfactory bulb of rats is excitatory in nature (Goldmakher and Moss, 2000). This led us to investigate the role of EXP-1 in chemotaxis/olfaction. Mutants of *exp-1* when tested in chemotaxis assays were specifically defective toward AWC-sensed odors. At the molecular level, neurons sense cues through G-protein-coupled receptors (GPCRs) present on their surface (Troemel et al., 1995; Sengupta et al., 1996). The expression of multiple olfactory GPCRs in the ASI chemosensory neurons (SRA-6, STR-2, STR-3, STR-1, SRD-1) is modified on exposure to pheromones and during dauer stages of development through DAF-7/TGF-β (Nolan et al., 2002; Lans and Jansen, 2006). The STR-2 receptor normally expressed in the AWC neuron is upregulated in the ASI neuron during dauer stages or in the absence of DAF-7/TGF-β signaling (Peckol et al., 2001). Transition in receptor levels affect neuron morphology and alter behaviors (Peckol et al., 2001; Dixit et al., 2020). To investigate the receptor-dependent developmental plasticity of the organism, we studied the role of STR-2 and EXP-1 in dauer formation. We show that STR-2 and EXP-1 in the ASI neuron sense the availability of food and promote reproductive stages in the absence of DAF-7/TGF-β. Further, simultaneous loss of *str-2* and *exp-1* in the *daf-7* mutant background progressed the animals into reproduction defective dauer stages.

## Materials and Methods

### *C. elegans* strains and maintenance

All *C. elegans* strains were maintained at 20°C on nematode growth media (NGM) plates seeded with *Escherichia coli* OP50 (Brenner, 1974). The strains used in this study are compiled in Table 1. All the strains were outcrossed at least three times. The *exp-1* mutant strain EG276 was outcrossed frequently (once every three to four months). N2 Bristol was used as the wild-type (WT) reference strain. Double or triple mutants were generated by standard genetic crosses and verified by PCR or fluorescence expression. The primers used for genotyping during outcrossing and for the generation of double or triple mutants are listed in Table 2.

**Table 1:** List of strains used in this study

Strain	Genotype	Comments
EG276	*exp-1(ox276) II*	3× out crossed (CGC strain)
RB2302	*Daf-7(ok3125 )III*	3× out crossed (CGC strain)
VC3044	*dbl-1(ok3749) V*	3× out crossed (CGC strain)
VC2413	*str-2(ok3089) V*	3× out crossed (CGC strain)
RB2464	*tax-2(ok3403 )I*	3× out crossed (CGC strain)
VC3113	*tax-4(ok3771) III*	3× out crossed (CGC strain)
FK311	*ceh-36(ks86) X*	3× out crossed (CGC strain)
FK181	*ksIs2[Pdaf-7::GFP + rol-6(su1006)]*	3 × out crossed (CGC strain)
CX3695	*Pstr-2*::GFP(kyIs140)	CGC strain
BAB1415	*exp-1(ox276); str-2 (ok3089)*	This study
BAB1421	*exp-1(ox276); daf-7(ok3125); str-2(ok3089)*	This study
BAB1424	*exp-1(ox276); daf-7(ok3125)*	This study
BAB0881	*exp-1(ox276); ceh-36(ks86)*	This study
BAB0882	*tax-2(ok3403; exp-1(ox276)*	This study
BAB0880	*exp-1(ox276); tax-4(ok3771)*	This study
BAB1577	*daf-7(ok3125); str-2(ok3089)*	This study
BAB1588	*exp-1/Pexp-1::*EXP-1in pPD95.75 *(indEx892)*	This study
BAB1589	*WT/Pexp-1::*EXP-1::sl2::wrmScarlet *(indEx893)*	This study
BAB1590	*exp-1/Pexp-1::*EXP-1::sl2::wrmScarlet *(indEx894)*	This study
BAB1591	*exp-1/ Pgpa-4::*EXP-1::sl2::wrmScarlet *(indEx895)*	This study
BAB1592	*(indEx894)/*P*srb-6*::GFP *(indEx896)*	This study
BAB1593	*exp-1;str-2/Pgpa-4*::STR-2::sl2::wrmScarlet *(indEx897)*	This study
BAB1594	*exp-1;str-2/Pstr-2*::STR-2::sl2::wrmScarlet *(indEx898)*	This study
BAB1595	kyIs140/P*daf-1*::sl2::wrmScarlet *(indEx899)*	This study

**Table 2:** List of oligonucleotides used in this study

Primer code	Sequence	Comment	Gene
PRS 40	CGT GGC GAG ACC CTC GAC	Forward external	*exp-1*
PRS 41	CTC GAA CGT AGC CGC CAA TTC	Forward internal	*exp-1*
PRS 42	GTG CCA ACT TTA TCA GGG AGA G	Reverse external	*exp-1*
PRS 439	AGGACGGAAATTACCTGTGC	Forward external	*daf-7*
PRS 440	GCTTCGGGAAACGCTCATAT	Reverse external	*daf-7*
PRS 441	TTATTCTTTCTTGTCGGGGCC	Reverse internal	*daf-7*
PRS 362	CTATTGCTTGCGCTCATAGGAAG	Genotyping forward external	*str-2*
PRS 363	CTTCAGTCATCATGTTGCACAATTTC	Genotyping reverse external	*str-2*
PRS 364	GTGTAATGTGGATTATACTTGTCAG	Genotyping reverse external	*str-2*
PRS 426	AGCTGTTGTCTCCTTCCAGG	Genotyping forward WT	*tax-2*
PRS 427	CGATTTCCGATGAGGAAACC	Genotyping forward mutant	*tax-2*
PRS 428	CACAGCTTCTAATAGGAAAGGG	Genotyping reverse Common	*tax-2*
PRS 394	GCGGTTCGGATACGAAAATACTTG	Genotyping forward external	*tax-4*
PRS 395	GACGGAGAAGTGTATCCGTTATATC	Genotyping reverse internal	*tax-4*
PRS 396	CCATGCGTCCGTCCCTAATCC	Genotyping reverse external	*tax-4*
PRS 403	GTGTGGTACCCAAGTTGATAGG	Genotyping forward common	*ceh-36*
PRS 404	GTTTTCCGCGAAACACAGTACC	Genotyping reverse WT	*ceh-36*
PRS 405	GTTTTCCGCGAAACACAGTACT	Genotyping reverse M	*ceh-36*
PRS 576	GGAGAGTCGTCATCGGCG	Genotyping forward external	*dbl-1*
PRS 577	GGCATTGGATTTGGACAAGAGC	Genotyping reverse external	*dbl-1*
PRS 578	CTGTGCAGACTGGTCCGAG	Genotyping forward internal	*dbl-1*
PRS 349	AAAACTGCAGcttgagagatccaatgaaatcgg	Cloning forward PstI	*exp-1* promoter in pPD95.75
PRS 351	CTAGTCTAGAgccatcaagttttggcag	Cloning reverse XbaI	*exp-1* promoter in pPD95.75
PRS 352	GGGGTACCatgtctgcatctattctaattttg	Cloning forward KpnI	*exp-I* *Genomic* in pPD95.75
PRS 353	CCGCTCGAGctagtagatgtcggcaaaccactc	Cloning reverse XhoI	*exp-I* *Genomic* in pPD95.75
PRS 369	ACATGCATGCgaacggtctgtgggctctgac	Cloning forward SphI	*str-2* promoter
PRS 370	AAAACTGCAGgcagacccatatgtgtgcacaaac	Cloning reverse PstI	*str-2* promoter
PRS 515	ACATGCATGCgaagccttgtttgtataagaaacgctg	Cloning forward SphI	*gpa-4* promoter
PRS516	CCCCCCGGGGttgaaaagtgttcacaaaatgaataagtg	Cloning reverse XmaI	*gpa-4* promoter
PRS 561	CACTTTTCAACGGatccccgggatgccgactgtgcaatgg	Cloning forward XmaI	*str-2* genomic in a wrmScarlet vector
PRS 566	GGGGTACCgcttgcgctcataggaagag	Cloning reverse KpnI	*str-2* genomic in a wrmScarlet vector
PRS 534	ACATGCATGCcggtcaccattcccaaattcgc	Cloning forward SphI	*daf-1* promoter
PRS 535	GGGGTACCcttgcacaggtaccaatttatgatg	Cloning reverse KpnI	*daf-1* promoter

### Plasmid construction and germline transformation

Constructs used in this study were generated by standard molecular biological techniques (Russell, 2001). Plasmids used to generate the rescue constructs were pPD95.75, pPD49.26 or the wrmScarlet vector (P*rig-3*::NPR-4::*sl2*::wrmScarlet) that was kindly provided by the Cori Bargmann lab. Germline transformation of the constructs was performed by microinjection as previously described (Mello et al., 1991; Mello and Fire, 1995). The promoter fusion constructs for expression and gene rescuing constructs were injected at 20–30 ng/μl. The constructs used in this study are tabulated in Table 3.

**Table 3:** List of plasmids used in this study

S. number	Plasmid number	Plasmid
1	pBAB0056	P*exp-1::*EXP-1in pPD95.75
2	pBAB0063	P*exp-1::*EXP-1::sl2::wrmScarlet
3.	pBAB0067	P*gpa-4::*EXP-1::sl2::wrmScarlet
4.	pBAB0069	P*gpa-4::*STR-2::sl2::wrmScarlet
5.	pBAB0070	P*str-2*::STR-2::sl2::wrmScarlet
6.	pBAB0071	P*daf-1*::sl2::wrmScarlet
7.	pBAB465	P*srb-6*::GFP (Kadam et al., 2021)

### Chemotaxis assay

To perform Chemotaxis assays, eggs were prepared and on hatching the *C. elegans* were grown till young adult stages. Assay was performed after 65 h (h) of incubation at 20°C. NGM plates w/o cholesterol were used for the assay. Wherever required, odorants were diluted in ethanol and reported as percent by volume. Modified 90 mm quadrant plate chemotaxis assays were performed as described previously (Bargmann et al., 1993; Margie et al., 2013). Plates were prepared a day in advance and dried for 1 h. Animals were collected using M9 buffer with 0.003% Tween 20 and washed three times with M9 buffer. Plates were divided into four quadrants and marked as control (C) and test (T) spots in adjacent sections and 3 cm from the loading center. To these spots, 2.0 μl of freshly prepared 1 m sodium azide (in M9) was added. Sodium azide acts as an anesthetic agent to immobilize *C. elegans* that reach the vicinity of the spot during the assay. After 2 min, animals (200–250) were added in the center of the plate. Once the *C. elegans* were ready on the assay plate, we added ethanol (3.0 μl) to the control spots and odors (3.0 μl) to the test spots. Plates were incubated for 90 min undisturbed and immediately after that moved to a 4°C refrigerator. Animals in the respective quadrants were counted after 3–4 h, and chemotaxis indices were calculated using the previously published formula; chemotaxis index = (number of animals in both test quadrants – number of animals in both control quadrants)/(total number of animals scored; Margie et al., 2013). Graphs of chemotaxis indices were then plotted. In case where animals were dauer, to get rid of dauer animals while washing, the adult animals were allowed to settle down and dauers were removed from the supernatant during a washing step. We counted only the *C. elegans* that moved from the center of the plate. WT animals showed a chemotaxis index of ∼0.7 for attractive odors and animals with chemotaxis defects showed lower or higher values.

### Dauer assay

Freshly prepared eggs were transferred to 60-mm NGM plates. Each plate was plated with ∼250–300 eggs. Plates were incubated at upto three different temperatures; 16°C, 20°C, and 25°C. Animals were allowed to grow, and once the *C. elegans* reached the adult stage, the animals were counted. Animals took variable times to grow at different temperatures. The timeline that was followed for scoring were ∼60 h for 25°C, ∼70 h for 20°C plates, and the 16°C plates were scored for dauer animals after 92 h (Ailion and Thomas, 2000). Graphs were plotted for the dauer phenotype as the percentage of dauers in the total population of *C. elegans*.

### Microscopy

Fluorescence imaging was performed on live animals. Briefly, animals were immobilized using 30 mg/ml 2,3-butanedione monoxamine (Sigma-Aldrich) prepared in M9 as previously described (Sieburth et al., 2005). In order to visualize and image, the animals were immobilized with 30 mg/ml BDM on 2% agarose pads (prepared in M9); 1 μm (total depth) Z-series stacks were collected using a Carl Zeiss fluorescence microscope Axio Imager Z2 with the Axiocam MRm camera equipped with GFP and red fluorescent protein filters. Images were collected and analyzed using the FIJI image J software (Schindelin et al., 2012). Maximum intensity projections of Z-series stacks were used for the analyses of expression and localization of fluorescent markers. The exposure settings were kept identical for all images taken in a single experiment. Imaging was done in the head region of the animals and 25–30 animals were imaged for each experiment. To image AWC and ASI cilia, imaging was performed using the Leica TCS SP8 confocal microscope, using the argon laser at 10% gain.

### Tracking of *C. elegans*

We recorded the chemotaxis behavior of animals for 90 min using a 5-megapixel CMOS USB camera (Mightex) and used the Mightex Camera Demo v1.1.0 software. Recordings were done in a Peltier cooled incubator at 20°C at 0.5 frames/s (Dahiya et al., 2019). Tracks of the animals were analyzed using the FIJI software (Schindelin et al., 2012).

### Statistical analysis

To perform statistical analysis, GraphPad Prism version 6.0 was used. The error bars represent SEM calculated for chemotaxis and dauer assay data. Statistical comparisons were done using one-way ANOVA with the Bonferroni multiple comparison test. The level of significance was set as *p < 0.05, **p < 0.01, and ***p < 0.001.

## Results

### Mutants in *exp-1* show defective response toward AWC-sensed odors

**Figure 1.**
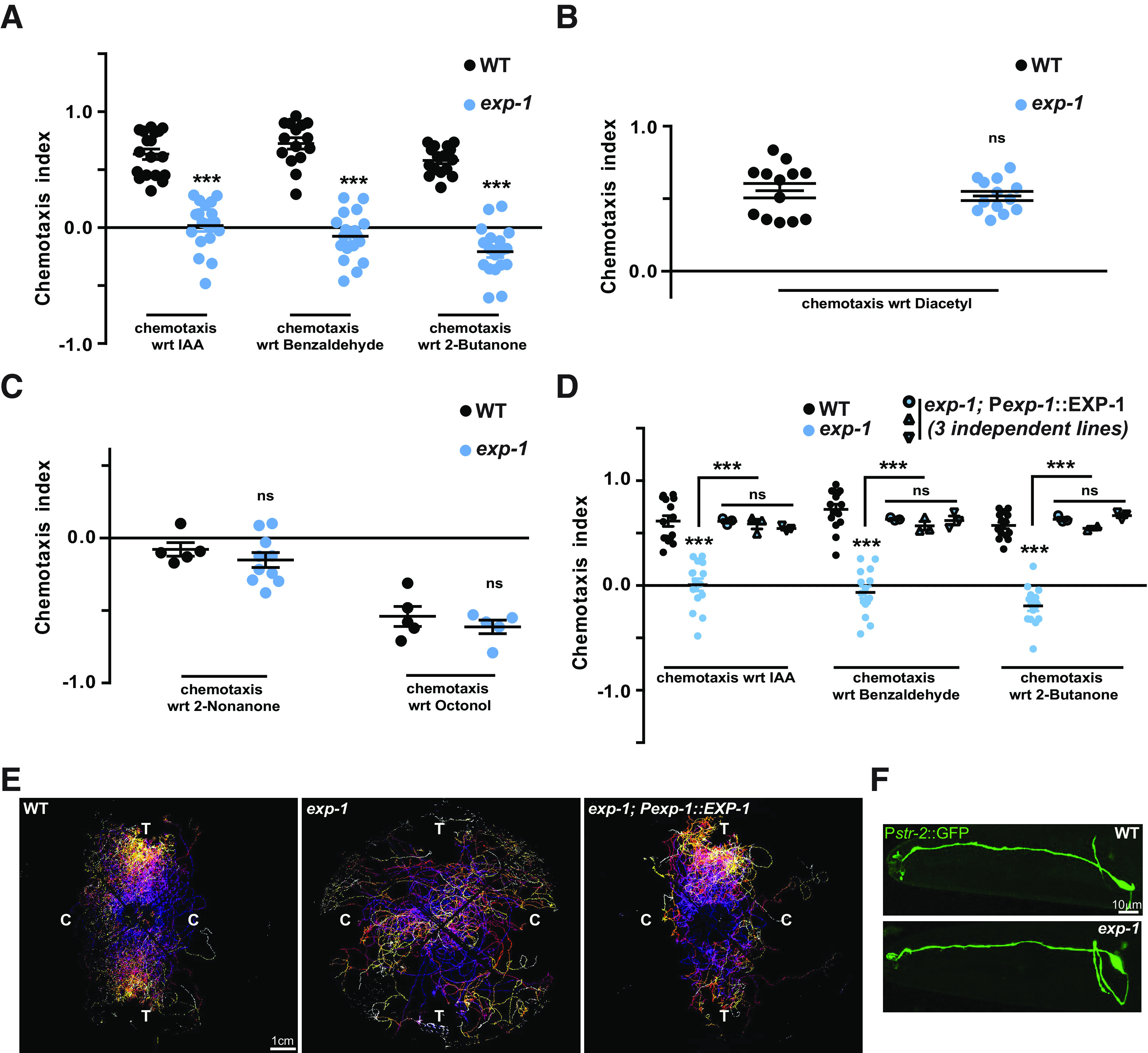
*exp-1* mutants show defects in AWC-dependent chemosensation. ***A***, Graph indicating chemotaxis indices for AWC-sensed attractants, IAA, benzaldehyde, and 2-butanone used at 1:1000 dilution for WT and *exp-1* animals. ***B***, Response of WT and *exp-1 C. elegans* toward the AWA-sensed attractant diacetyl (1:1000). ***C***, Avoidance response of *exp-1* and WT animals toward the AWB-sensed odors, Nonanone and 1-octanol (1:100 dilution for both). Each circle in the graphs represents one assay performed using ∼200–250 *C. elegans* for all graphs from ***A–C***. ***D***, Defects in *exp-1* mutants were rescued using the endogenous *exp-1* promoter. Each circle or triangle in the graph represents one assay performed using ∼200–250 *C. elegans*. ***E***, Tracks of WT (left panel), *exp-1* (middle panel), and the *exp-1* rescuing line (right panel) animals during chemotaxis toward IAA. ***F***, Fluorescent confocal images of the AWC neuron of WT and *exp-1* mutants expressing P*str-2*::GFP. In all graphs, the error bars represent SEM; *p* values are indicated as ***p < 0.001; ns, not significant in all graphs based on *p* values calculated using one-way ANOVA with Bonferroni multiple comparison test.

It has been previously reported that EXP-1 is expressed in a few head neurons in addition to its expression in the enteric muscles of the intestine (Beg and Jorgensen, 2003). There were a pair of unidentified neurons that expressed EXP-1, and we were interested in delineating these neurons. The morphologic position of these neurons was evaluated by referencing to Worm Atlas. We suspected the neurons to be AWC neurons. To this end, we tested *exp-1* mutant animals for volatile odors sensed by the AWC neurons, using established chemotaxis assays (Bargmann and Horvitz, 1991). First, we tested for isoamyl alcohol (IAA), and found that the *exp-1* mutant animals were defective in recognizing this odor (Fig. 1*A*). To further investigate the olfactory defects of *exp-1* mutants we tested these mutant animals for other attractive volatile odors (benzaldehyde, 2-butanone) sensed by the AWC neuron. We found that *exp-1* animals were defective in the recognition of all the known attractive odors sensed by the AWC neuron (Fig. 1*A*). To understand whether this behavior was specific for AWC-sensed volatile odors, we tested *exp-1* mutant animals for an AWA-sensed attractive odor, diacetyl and found that these animals behaved like WT control animals in the presence of diacetyl (Fig. 1*B*). Next, we tested *exp-1* mutants against the repulsive volatile odors, nonanone and 2-octanol that are sensed by the AWB neuron. Mutants in *exp-1* were indistinguishable from WT animals in their repulsion to nonanone and 2-octanol (Fig. 1*C*). Our data suggest that *exp-1* mutant animals were defective only for AWC-sensed odors. We next generated a rescue construct by using the endogenous *exp-1* promoter to express EXP-1. Chemotaxis assays were performed, and we observed that expressing EXP-1 under its native promoter in *exp-1* mutant animals rescued the chemotaxis defects seen in the mutant *C. elegans* (Fig. 1*D*). We also wanted to understand the movement of animals during chemotaxis and tracked WT, *exp-1* mutants, and *exp-1* mutants containing the rescue construct during the chemotaxis assay. Our data showed that in case of WT and the *exp-1* rescue lines, the animals very quickly oriented in the direction of the attractant and moved to the quadrants containing the attractants (Fig. 1*E*, left and right panels). The *exp-1* mutant animals however moved all over the plate and a majority of these *C. elegans* localized to the control quadrants showing that they were not attracted to AWC-sensed attractants (Fig. 1*E*, middle panel). Previous studies have shown that defects in the AWC cilia, that are the sites of odor perception, can lead to defective responses toward odors (O’Halloran et al., 2009). We speculated that there could be defects in the cilia of AWC neurons in *exp-1* mutants, and this might explain the defects toward AWC-sensing odors. We imaged and analyzed the AWC neurons of control and *exp-1* mutant animals and found no obvious difference in the morphology of the AWC neurons or their cilia (Fig. 1*F*). These results suggest that EXP-1 participates in AWC neuron-dependent chemotactic behaviors, although *exp-1* mutants show no significant defects in the AWC neuron morphology.

### EXP-1 is expressed and functions in the ASI chemosensory neuron

**Figure 2.**
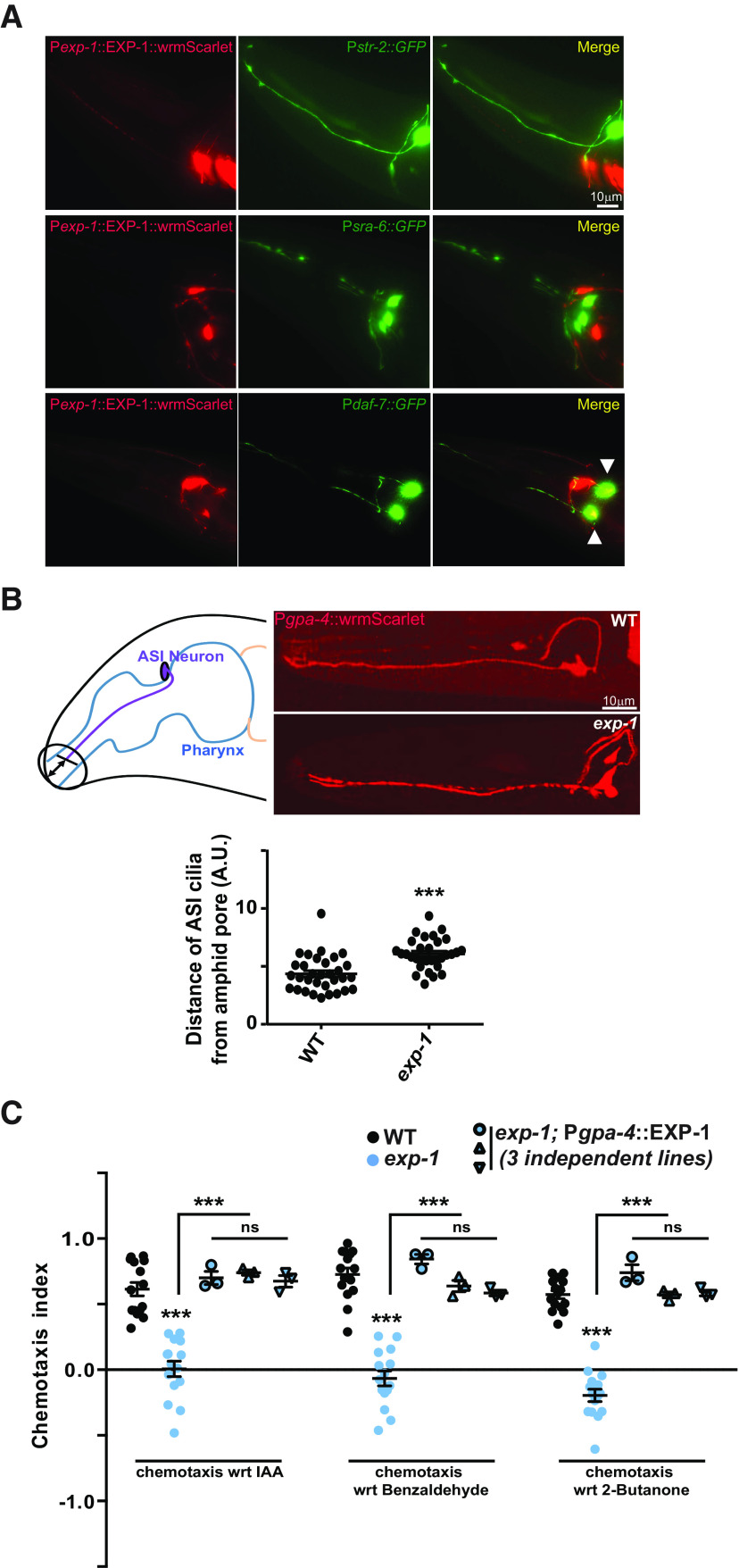
EXP-1 is expressed in the ASI amphid neuron and regulates AWC-dependent chemotaxis in a non-cell autonomous manner. ***A***, Localization of P*exp-1*::wrmScarlet with respect to the AWC neuron marked with GFP (top panel), ASH neurons marked with GFP (middle panel), and the ASI neurons marked with GFP (bottom panel). ***B***, Illustration of the ciliary region of the ASI neuron toward the amphid pore (left panel). Confocal images of the ciliary region of the ASI neuron from WT (upper right panel) and *exp-1* mutants (lower right panel). The bottom panel indicates a graph depicting the distance of the ciliary end from the externally exposed end of the amphid pore as shown in the illustration. This was measured as an arbitrary unit (A.U.) by using the analysis tool of FIJI. Each circle corresponds to one animal. ***C***, AWC-dependent chemotaxis defect of *exp-1* mutants were rescued by ASI-specific expression of EXP-1. Each circle or triangle in the graph represents one assay performed using ∼200–250 *C. elegans*. Error bars represent SEM; *p* values are indicated as ***p < 0.001; ns, not significant in all graphs based on *p* values calculated using one-way ANOVA with Bonferroni multiple comparison test.

In order to understand the neuronal basis of *exp-1* mutant defects, we studied the expression pattern of EXP-1. To study the expression of EXP-1, we generated a P*exp-1::*EXP-1::SL-2::wrmScarlet expression line. Upon imaging we observed that as reported previously, there was expression in the enteric muscles and in the head neurons (Fig. 2*A*; Beg and Jorgensen, 2003). Since we found AWC-dependent phenotypes in *exp-1* mutants, we used the AWC-expressing *str-2::GFP* marker line and studied its co-localization with P*exp-1*::EXP-1::sl2::wrmScarlet. Surprisingly, our imaging results revealed that EXP-1 was not expressed in the AWC neurons (Fig. 2*A*, upper panel). Since *exp-1* mutants showed aversive behavior toward attractants, we performed EXP-1 expression analysis in the ASH neurons (Troemel et al., 1995; Chao et al., 2004). We crossed an ASH neuron-specific marker line, P*srb-6*::GFP into the P*exp-1::*EXP-1::sl-2::wrmScarlet background and studied the localization of the two fluorescent markers. Our imaging analysis suggested that EXP-1 was not expressed in the ASH neurons (Fig. 2*A*, middle panel). Based on previous studies where EXP-1 was shown to participate in ASI-dependent behaviors (Bendesky et al., 2012), we studied EXP-1 expression in the ASI neurons. ASI neurons are amphid sensory neurons with direct links between the sensory input in the form of food cues and neuroendocrine signaling through the production of DAF-7/TGF-β (Golden and Riddle, 1982, 1984a; Nolan et al., 2002). For this experiment, a P*daf-7*::GFP marker line was introduced into the EXP-1 expression line. Our imaging results clearly revealed that EXP-1 was localized to the ASI neuron along with P*daf-7*::GFP (Fig. 2*A*, lower panel). ASI is a chemosensory neuron that belongs to the group of amphid neurons whose cilia are directly exposed to the outer milieu (Bargmann and Horvitz, 1991). It detects water-soluble chemicals and pheromones and is the major regulator of DAF-7/TGF-β-dependent dauer formation (Albert et al., 1981; Golden and Riddle, 1982, 1984a; Gottlieb and Ruvkun, 1994; Ren et al., 1996; Schackwitz et al., 1996). A previous study has shown that in dauer stages the cilia are retracted (Ward et al., 1975) and retracted cilia have been shown to sense volatile odors (Bargmann et al., 1993). In order to study the cilia in the ASI neurons, we performed imaging analysis of the ASI ciliary endings in WT and *exp-1* mutant animals. Confocal imaging was performed and the ciliary endings were measured from the extreme anterior end of animals (illustrated in Fig. 2*B*, left panel). Analyses of *exp-1* mutants (Fig. 2*B*, lower panel) when compared with the WT control animals (Fig. 2*B*, upper panel) revealed that in these mutants, the ASI cilia were significantly withdrawn from the amphid pore (Fig. 2*B*, bottom panel). Next, we expressed EXP-1 specifically in the ASI neurons using the *gpa-4* promoter (P*gpa-4::*EXP-1) and found that ASI expression of EXP-1 completely rescued the chemotaxis defects associated with the *exp-1* mutants (Fig. 2*C*). These rescue experiments revealed that the alteration in chemotaxis behavior of *exp-1* was also ASI dependent. Together, these data revealed that EXP-1 is expressed and appears to function in the ASI neurons to regulate AWC-dependent chemotaxis.

### EXP-1 participates in AWC-dependent chemotaxis in a non-cell autonomous manner

**Figure 3.**
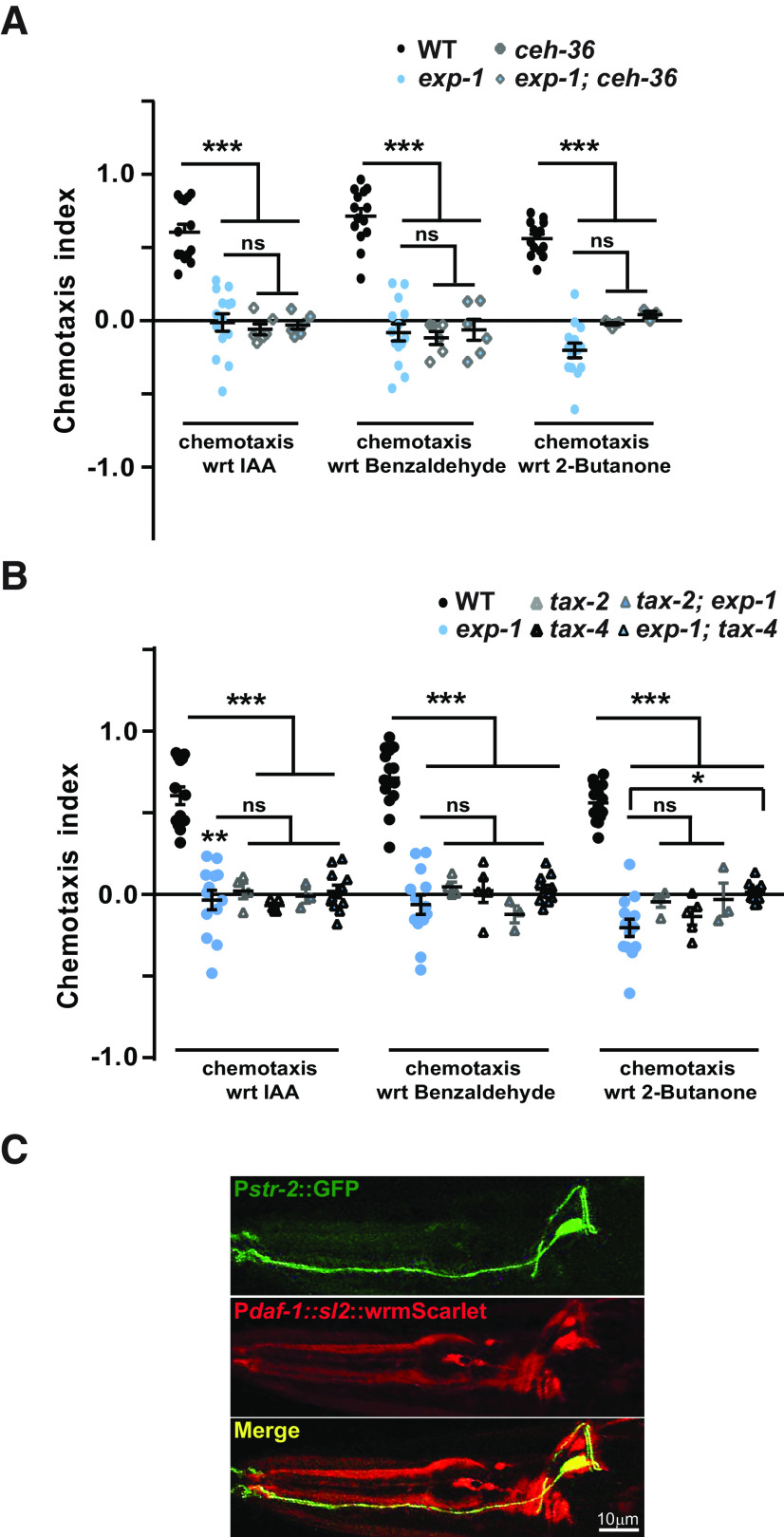
The chemotaxis defects of *exp-1* require a functional AWC neuron. ***A***, Graph indicating chemotaxis indices of *exp-1; ceh-36 C. elegans* along with control animals. ***B***, Graph indicating chemotaxis indices of *tax-2; exp-1* and *exp-1; tax-4 C. elegans* along with control animals. Each circle, quadrilateral, or triangle in the graphs represents one assay performed using ∼200–250 *C. elegans*. Error bars represent SEM; *p* values are indicated as ***p < 0.001, **p < 0.001, and *p < 0.05 in all graphs based on *p* values calculated using one-way ANOVA with Bonferroni multiple comparison test. ***C***, Fluorescence images of AWC neuron showing co-expression of STR-2 (top panel) and DAF-1 proteins (middle and bottom panels).

As detailed above, *exp-1* mutants are defective for AWC-sensed odors. We next wanted to examine the role of the AWC neuron in this behavioral defect. First, we asked what would be the response of *exp-1* mutants toward odor detection if AWC sensory neurons were not functional. Here, we used a mutant of *ceh-36.* CEH-36 is an OTX/OTD transcription factor and plays a role in the terminal differentiation of AWC neurons. Loss of *ceh-36* results in the loss of chemosensation toward AWC-sensed odors (Lanjuin et al., 2003). When we assayed the double mutants of *ceh-36; exp-1*, it showed defects similar to those seen in *ceh-36* mutant animals (Fig. 3*A*).

Next, we examined the primary AWC chemosensory transduction genes *tax-2* and *tax-4* that encode cyclic nucleotide gated channels, which transduce signals sent by GPCRs such as the *str* genes (Coburn and Bargmann, 1996; Komatsu et al., 1999; Cho et al., 2004, 2005). Notably, they are required in AWC neurons for chemosensory signal transduction and mutation in these genes lead to defects in AWC-dependent chemosensory responses (Fig. 3*B*; Bargmann et al., 1993; Chalasani et al., 2007). We generated double mutants of *exp-1* with both *tax-4* and *tax-2*, respectively. Our results demonstrated that double mutants generated with these signal transduction genes were also defective in chemotaxis, and showed behaviors similar to that seen in the *tax-4* and *tax-2* single mutants (Fig. 3*B*). These results demonstrate that the chemotaxis defects in *exp-1* mutants are similar to the defects observed because of defective AWC neurons and AWC developmental and signaling components did not show additive defects with *exp-1* mutant animals. Our data thus far suggest that although EXP-1 is expressed in the ASI chemosensory neurons, it appears to be required for the chemosensory function of the AWC neurons.

To understand the non-cell autonomous function of EXP-1, we wanted to understand the connectivity between ASI and AWC and found that ASI makes chemical synapses with the AWC neuron (*Wormatlas*; Oshio et al., 2003). The ASI neuron secrete small molecules like neuropeptides, insulin like peptides and/or DAF-7/TGF-β that could aid in the cross talk between ASI and AWC. DAF-7 expression was shown to be regulated by EXP-1 (Bendesky et al., 2012), and we hypothesized that it might be through DAF-7 that EXP-1 is regulating AWC function. DAF-7 signals are transduced through its widely expressed receptor components DAF-1 and DAF-4 that function as heterotetramers (Patterson et al., 1997; Gunther et al., 2000; Inoue and Thomas, 2000; da Graca et al., 2004). We speculated that these receptors may be present on AWC neurons which would then receive DAF-7 signals from ASI neurons. We chose to study DAF-1 receptor as DAF-4 also binds with another TGF-β protein DBL-1. To study DAF-1 expression in the AWC neuron we transformed DAF-1::WrmSc into the AWC reporter (STR-2::GFP) line and performed imaging experiments. Our results clearly showed that DAF-1 localizes to the AWC neuron (Fig. 3*C*). Hence, the AWC neuron could receive DAF-7 signals from the ASI neuron. This hypothesis is also supported by our previous results where we found that expression of EXP-1 in the ASI neuron fully rescued the chemotaxis defect of *exp-1* mutants (Fig. 2*B*). Thus, EXP-1 appears to be functioning in a non-cell autonomous manner to regulate AWC-mediated chemotaxis toward attractants and the cross talk could be occurring through DAF-7/TGF-β signaling.

### The chemotaxis defects of *exp-1* mutants are mediated through DAF-7/TGF-β and STR-2

**Figure 4.**
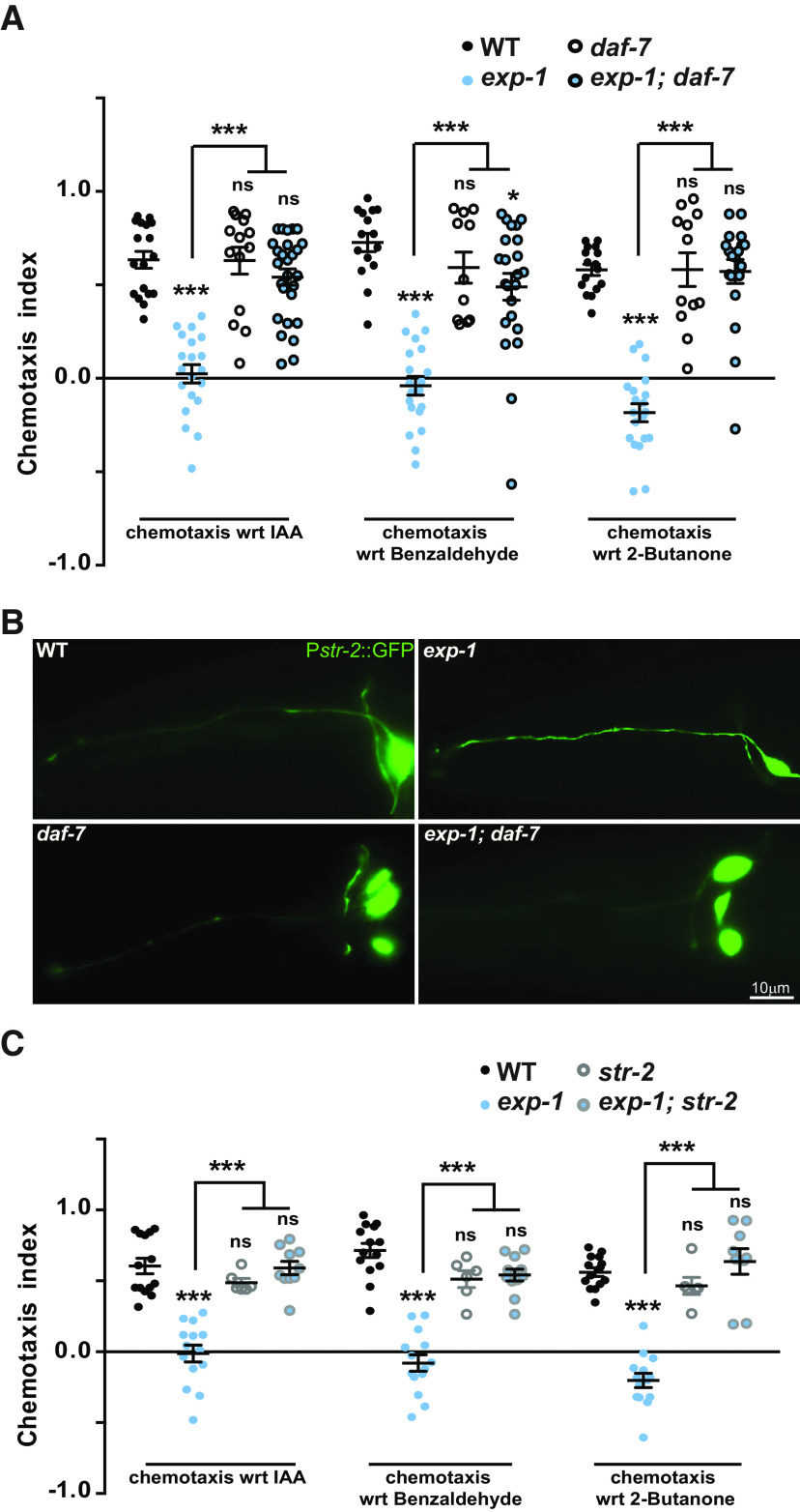
Mutants in *daf-7* suppress the *exp-1* mutant phenotype. ***A***, Graph indicating chemotaxis indices of *exp-1; daf-7* animals along with control *C. elegans*. ***B***, P*str-2*::GFP expression in *exp-1; daf-7* mutants along with WT and single mutant animals. ***C***, Graph indicating chemotaxis indices of *exp-1; str-2 C. elegans* along with control animals. Each circle in graphs ***A***, ***C*** represents one assay performed using ∼200–250 *C. elegans*. Error bars represent SEM; *p* values are indicated as ***p < 0.001, *p < 0.05; ns, not significant in all graphs based on *p* values calculated using one-way ANOVA with Bonferroni multiple comparison.

In the ASI neuron, DAF-7/TGF-β pathway components regulate behaviors that are directly dependent on the sensory capabilities of the animals and display appropriate physiological behaviors like aggregation, dauer formation, etc. (Ren et al., 1996; Riddle and Albert, 1997; Peckol et al., 2001; Nolan et al., 2002; Gallagher et al., 2013). It has been reported previously that in the absence of *exp-1, daf-7* levels are also reduced (Bendesky et al., 2012). We tested the involvement of *daf-7* in *exp-1* function by performing chemotaxis assays using the *exp-1; daf-7* double mutants. Our results showed that *daf-7* mutants are not defective in their response toward AWC-dependent odors. However, *daf-7* mutations completely suppressed the *exp-1* mutant phenotype and the double mutants behaved like WT control animals (Fig. 4*A*). Next, we asked how *daf-7* loss might be suppressing the *exp-1* phenotype. One possibility was change in expression of GPCRs regulated by DAF-7/TGF-β, present on the cilia for the detection of odors (Nolan et al., 2002). STR-2 is a GPCR serpentine receptor, normally expressed in one of the two AWC neurons and shows low/no expression in ASI in WT conditions. STR-2 expression in the ASI neuron is increased in the absence of *daf-7* or during conditions that result in ASI-dependent behaviors (Peckol et al., 2001; Nolan et al., 2002). Expression shift of STR-2 is interesting since no receptor related function is reported for this developmental shift. It has been shown that DAF-7/TGF-β genetically interacts with EXP-1 (Bendesky et al., 2012). To probe the possibility that loss of *exp-1* might also be able to affect the STR-2 expression pattern like DAF-7/TGF-β, we examined the expression pattern of STR-2 in the *exp-1* mutant background. Our imaging results revealed that *exp-1* mutants did not show obvious defects in STR-2::GFP expression, it was expressed unilaterally in the AWC neuron similar to what was seen in WT control animals (Fig. 4*B*, upper panels). As reported previously, we also found that *daf-7* mutants showed upregulated expression of STR-2 in the ASI neuron (Fig. 4*B*, lower left panel). Our imaging results for *exp-1*; *daf-7* double mutants showed that here too the expression pattern was altered for STR-2, and it was expressed in both the ASI and AWC neurons similar to what was seen in *daf-7* mutants (Fig. 4*B*, lower right panel). These data suggested that *exp-1* does not affect the STR-2 expression levels in the ASI neurons. We next went on to study the role of *str-2* mutants in conjunction with *exp-1*. We tested *str-2* mutant animals in chemotaxis assays and found that they were not defective in chemotaxis toward AWC-sensed odors. Next, we tested the *exp-1; str-2* double mutants and found that these animals behaved like *str-2* single mutants (Fig. 4*C*). Thus, the *exp-1* defects toward AWC-sensed odors require a functionally WT form of STR-2 which is likely to function as a receptor as previously shown for 2-heptanone (Zhang et al., 2016). We also investigated whether STR-2 was functioning in ASI or AWC neurons to participate in *exp-1* mutant chemosensory defects by using the *gpa-4* and *str-2* endogenous promoters to drive STR-2 expression in the ASI and AWC neurons, respectively. These rescue constructs were transformed into *exp-1; str-2* double mutants and studied for their chemotaxis responses Quantitative analysis of chemotaxis revealed that STR-2 when expressed in the AWC but not in the ASI neurons could partially recapitulate the *exp-1* mutant behaviors (Extended Data Fig. 4-1*A*,*B*). These results indicate that the *exp-1* mutant behavior is dependent on ASI secreted DAF-7 and AWC located STR-2.

10.1523/ENEURO.0473-20.2021.f4-1 Extended Data Figure 4-1 STR-2 functions in the AWC neurons to participate in *exp-1* mutant behavior. **A**, Graphs for *exp-1; str-2* strain transformed with STR-2 gene expressed under the ASI-specific promoter *gpa-4* along with controls. ***B***, Graphs for *exp-1; str-2* strain transformed with STR-2 gene expressed under the AWC-specific promoter *str-2* along with controls. Error bars represent SEM; ***p < 0.001, **p < 0.01, *p < 0.05, ns, not significant in all graphs based on *p* values calculated using one-way ANOVA with Bonferroni multiple comparison test where relevant. Download Figure 4-1, EPS file.

### STR-2 and EXP-1 also regulate dauer formation

**Figure 5.**
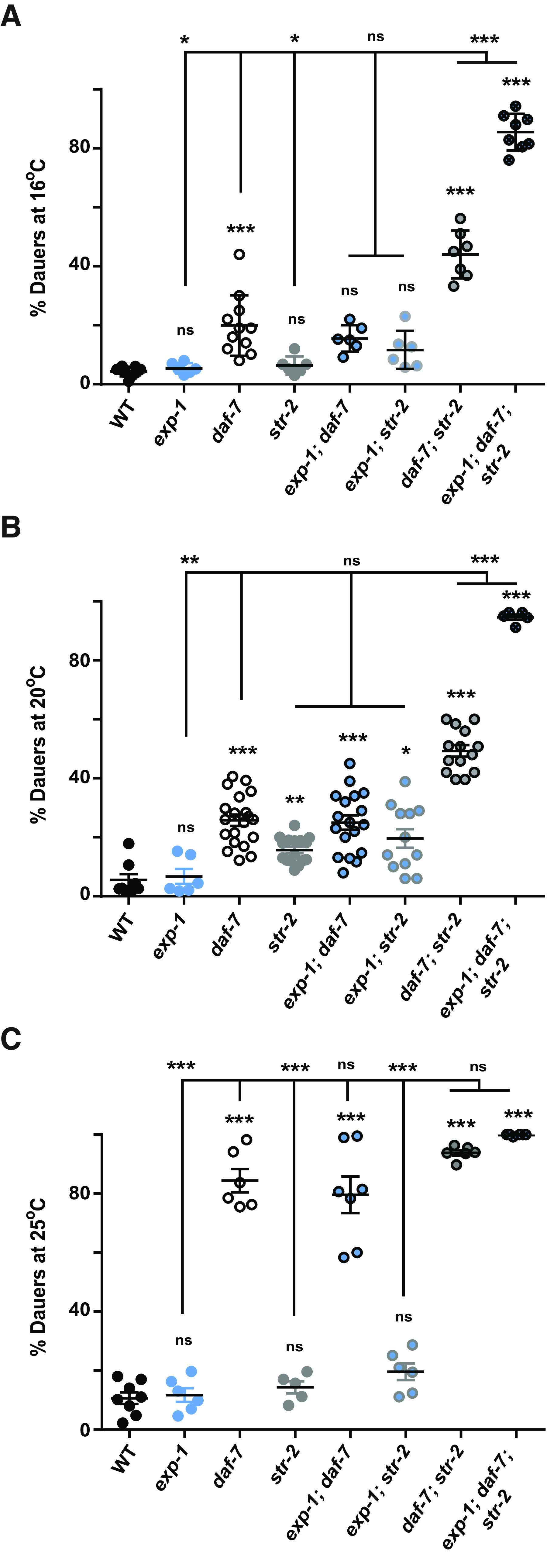
Loss of *str-2* in the *daf-7* mutant background leads to an enhanced dauer phenotype that is further enhanced in the absence of *exp-1*. Dauer assays were performed for the *exp-1; daf-7; str-2* mutant animals along with WT, single mutant, and double mutant *C. elegans*. The assay was performed at three temperatures: (***A***) 16°C, (***B***) 20°C, and (***C***) 25°C. Each circle in the graphs represents a single experiment performed using ∼200–300 animals per plate. Error bars represent SEM; *p* values are indicated as ***p < 0.001, **p < 0.01, *p < 0.05; ns, not significant in all graphs based on *p* values calculated using one-way ANOVA with Bonferroni multiple comparison test where relevant.

Based on previous work and our studies it is evident that EXP-1 and STR-2 function in ASI amphid neurons and can regulate behaviors through DAF-7/TGF-β. Both ASI neurons and DAF-7/TGF-β affect dauer formation (Thomas et al., 1993; Peckol et al., 2001; Cheung et al., 2004; Shaw et al., 2007). This got us interested in studying the role of STR-2 and EXP-1 in dauer formation. To test this, we performed dauer assays using *exp-1*, *daf-7*, and *str-2* mutants as controls along with their double and triple mutants, at temperatures of 16, 20 and 25°C. We observed that *str-2* mutant animals did not show differences in the percentage of dauers when compared with WT animals at 16°C, but dauer formation was slightly, albeit significantly higher at 20°C. Next, we generated *daf-7; str-2* double mutants, that were difficult to obtain and involved screening through hundreds of heterozygous animals. Surprisingly, the double mutants showed significant increase in dauers when compared with either of the two single mutant animals at 16°C and 20°C (Fig. 5*B*). Thus, our dauer experiments reveal that STR-2 might be responding to changes in environmental conditions in the absence of *daf-7* through alternate pathways. However, double mutants of *daf-7; str-2* are not completely dauer, suggesting that there could be molecules still sensing environmental signals in these mutant animals. Since *exp-1* is also a receptor, we explored the possibility of its role in sensing the environment and in maintaining the reproductive phase versus entry into the dormant dauer stage. Dauer quantitation showed that *exp-1* mutants by themselves behaved like WT control animals and *exp-1; daf-7* double mutants behaved like *daf-7* mutants. We also tested double mutants of *exp-1; str-2* and found that they showed a small but significant increase in the dauer phenotype at 20°C. Next, we generated triple mutants where *exp-1*, *daf-7*, and *str-2* were simultaneously removed. Surprisingly, we found that these animals showed close to 80% dauer phenotype even at 16°C and the severity increased to close to 100% dauers at higher temperatures. Given that EXP-1 and STR-2 are receptors are expressed in ASI and the increased dauer phenotype in the triple mutants was evident even at lower growth temperatures and in the presence of food, a plausible explanation for these results could be that EXP-1 and STR-2 sense environmental signals and modulate developmental balance toward reproductive phase through alternate pathway/s in the absence of DAF-7/TGF-β.

### STR-2 expression in ASI maintains reproductive growth in the absence of *daf-7*

**Figure 6.**
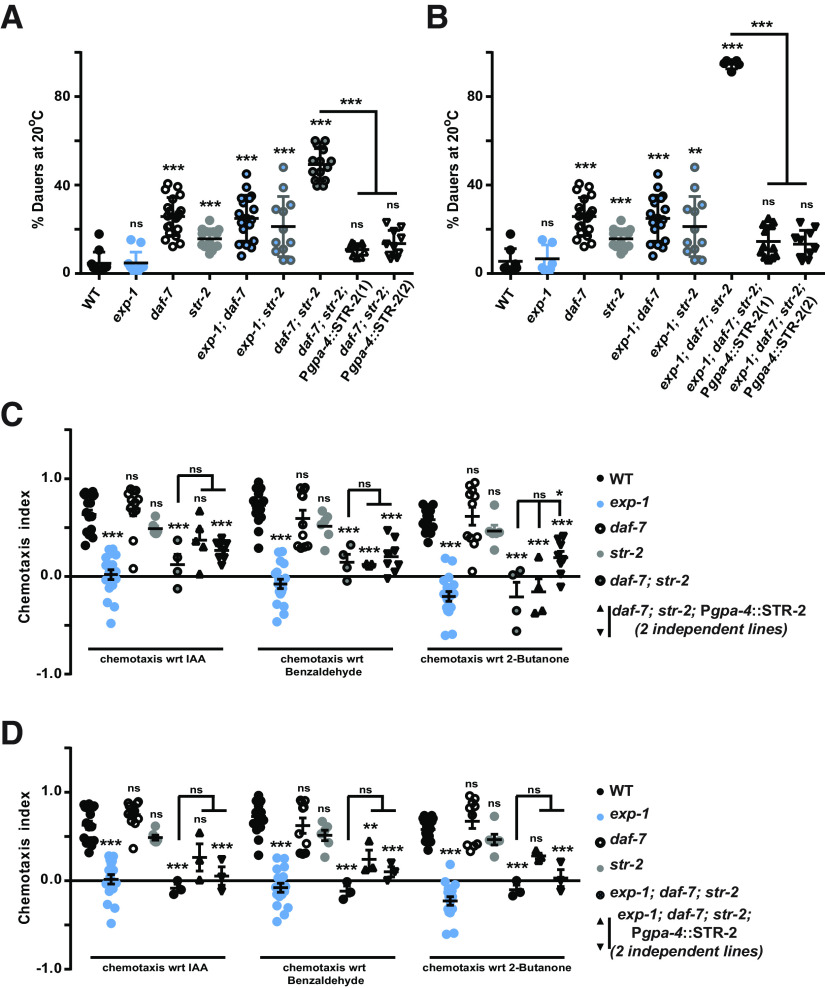
STR-2 expression in ASI neurons completely rescues the dauer phenotype and partially rescues the chemotaxis defects of *exp-1; daf-7; str-2* mutant animals. ***A***, STR-2 rescue of the dauer phenotype of *daf-7; str-2* mutant animals along with controls. This experiment was performed at 20°C. ***B***, STR-2 rescue of the dauer phenotype of *exp-1; daf-7; str-2* mutant animals along with controls. This experiment was performed at 20°C. ***A***, ***B***, Each circle or triangle in the graphs represents a single experiment performed using ∼200–300 animals per 60-mm plate. ***C***, Graph representing chemotaxis indices for STR-2 rescue assays performed in double mutant (*daf-7; str-2*) animals along with controls. ***D***, Graph representing chemotaxis indices for STR-2 rescue assays performed in triple mutant (*exp-1; daf-7; str-2*) animals along with controls. Each circle or triangle in graphs ***C***, ***D***, represents one assay performed using ∼200–250 *C. elegans*. Error bars represent SEM; *p* values are indicated as ***p < 0.001, **p < 0.01, *p < 0.05; ns, not significant in all graphs based on *p* values calculated using one-way ANOVA with Bonferroni multiple comparison test where relevant.

Our data so far suggest that STR-2 contributes to maintain reproductive growth phase in the absence of *daf-7*. Since in *daf-7* mutants, STR-2 expression increases in the ASI neuron, thus it is appropriate to assume that it acts as a receptor in this neuron for the perception of conditions favoring reproductive state over the dauer state. To confirm the function of STR-2 in the ASI neuron for dauer formation, we used the *gpa-4* promoter and expressed STR-2 (P*gpa-4::str-2*) in the ASI neuron. We transformed this construct in the dauer forming double (*daf-7; str-2*) and triple (*exp-1; daf-7; str-2*) mutant backgrounds. These dauer-forming animals showed a large recovery on STR-2 expression in the ASI neuron and appeared to behave like WT animals (Fig. 6*A*,*B*). These data further supported our hypothesis that STR-2 can function in ASI neuron by activating unknown alternate pathway/s. These results also suggest that in the absence of *daf-7*, STR-2 allows for sensing favorable conditions such as food.

The dauer phenotypes observed with *daf-7; str-2* double and *exp-1*; *daf-7; str-2* triple mutants were obvious even at 16°C in the presence of food, implying that sensory loss of olfaction might be the reason for this developmental defect. These findings further indicated that STR-2 and EXP-1 sense food cues in ASI neurons and the dauer phenotypes observed for these mutants could be largely because of the sensory defects. Thus, we next tested these double and triple mutants for chemotaxis toward IAA, Benzaldehyde and 2-Butanone. To perform chemotaxis assays, we specifically took the non-dauer animals as dauer animals are completely chemosensation defective (Bargmann and Horvitz, 1991). Non-dauer animals were collected by sedimentation that enriches for non-dauers (heavier animals). The lighter dauer animals were discarded. We found that the double mutants of *daf-7; str-2* were also defective in their response to AWC-sensed odors (Fig. 6*C*,*D*). Moreover, we found that the triple mutant dauer animals either did not move or moved toward the control odor. The ASI-specific rescue of STR-2 could modestly overcome the chemotactic defects in these mutants (Fig. 6*C*,*D*). In *daf-7* mutants, STR-2 is also expressed in AWC neurons along with its upregulation in ASI. Since the odors tested here were mainly detected through the AWC neuron, thus it indicates that chemotaxis defect of *str-2* could be because of changes in its expression pattern and also because of changes in animal physiology associated with dauer phenotype (Cassada and Russell, 1975). Taken together, our data suggest that EXP-1 and STR-2 contribute toward the maintenance of the reproductive state during development and may function in the absence of DAF-7/TGF-β through other signaling pathways.

### TGF-β molecule, DBL-1 also participates in chemosensation and modulates dauer formation

**Figure 7.**
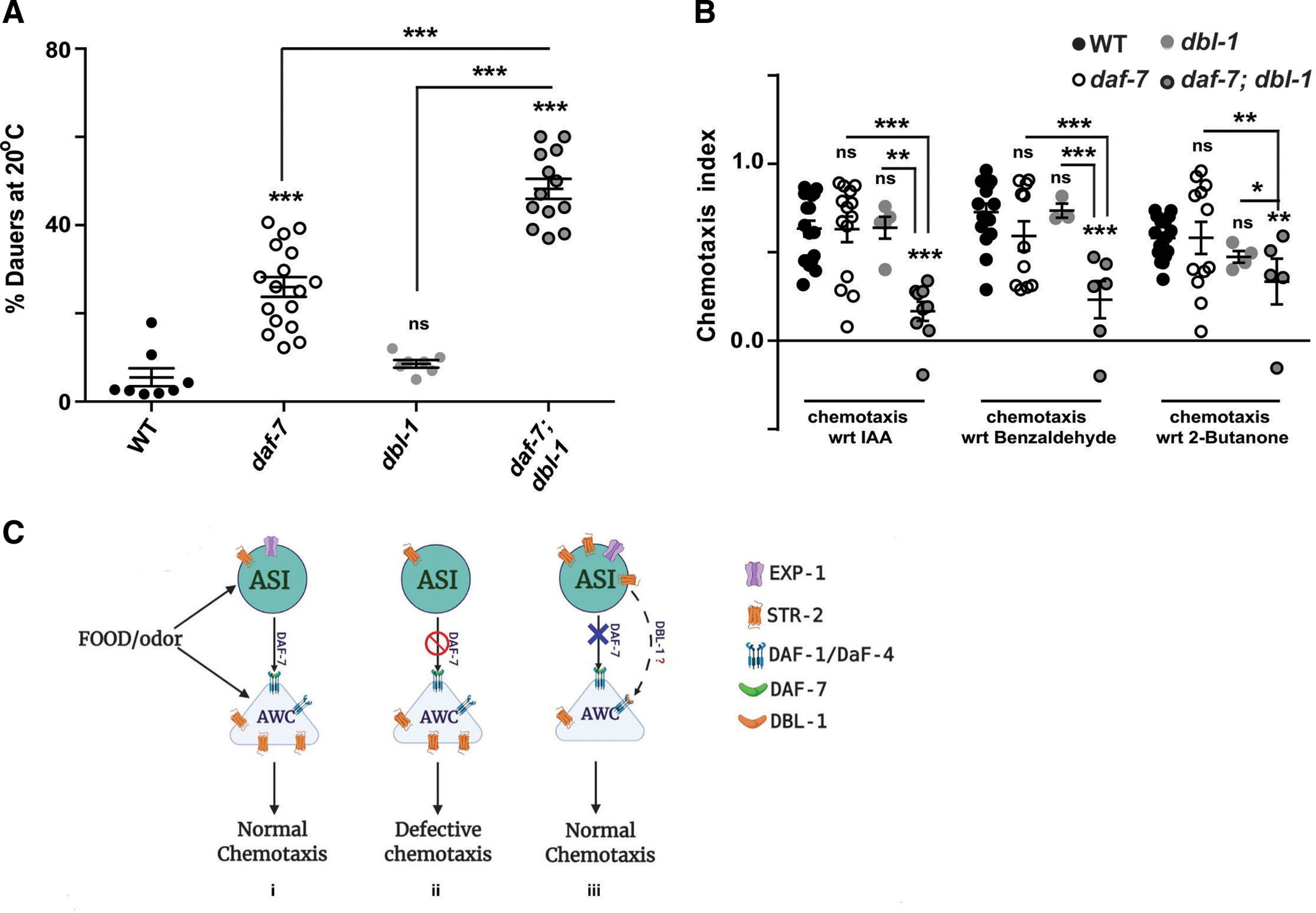
Mutants of *dbl-1* (TGFβ/BMP-like ligand) along with *daf-7* mutants resulted in dauer as well as chemotaxis defective phenotypes. ***A***, Graph indicating the dauer phenotype of *daf-7; dbl-1 C. elegans* along with control animals. This experiment was performed at 20°C. Each circle in the graphs represents a single experiment performed using ∼200–300 animals per 60-mm plate. ***B***, Graph indicating chemotaxis indices of *daf-7; dbl-1 C. elegans* along with control animals. Each circle in the graphs represents one assay performed using ∼200–250 *C. elegans*. Error bars represent SEM; *p* values are indicated as ***p < 0.001, **p < 0.01, *p < 0.05; ns, not significant in all graphs based on *p* values calculated using one-way ANOVA with Bonferroni multiple comparison test where relevant. ***C***, A predicted model for how AWC-dependent and ASI-dependent chemosensory cues could lead to dauer formation. ***i***, Normally AWC is responsible for sensing attractive odors and ASI senses food-dependent odors. They communicate with each other and maintain the reproductive phase of the organism. ***ii***, In the absence of *exp-1,* ASI neuron is able to sense the volatile odors because of retracted cilia. Here, levels of DAF-7 are low (EXP-1 regulates DAF-7 expression levels) and as a result the communication between AWC and ASI is aberrant. Moreover, ASI also has strong connections with downstream interneurons (AIB, AIY, AIA), which are normally involved in AWC chemosensory signaling leading to chemosensory bias between AWC and ASI neuron signaling and hence repulsion toward attractive odors. ***iii***, In the absence of *daf-7,* STR-2 expression in the ASI neuron is upregulated and our results indicate that both STR-2 and EXP-1 might be sensing food odors and communicating with the AWC neuron through an alternate pathway (possibly DBL-1 dependent). This could explain why double mutants of *daf-7* with *str-2*, *exp-1*, and *dbl-1* all show increased percentage of dauers as compared with *daf-7* single mutants. Moreover, triple mutants of *daf-7* with both *str-2* and *exp-1* mutations show an exaggerated dauer phenotype, possibly because of the loss of the alternate food sensory pathway (dashed line) in the absence of *daf-7*.

Previous reports indicate that another TGF-β molecule, DBL-1 also prevents entry into dauer phase (Roberts et al., 2010). DBL-1 is expressed in AFD amphid neurons in the head region in addition to other neurons. AFD neurons are temperature-sensing neurons, and it is possible that DBL-1 is regulating the dauer-reproductive switch based on temperature. Studies have shown that fluctuations in DAF-7/TGF-β levels are not temperature dependent (O’Donnell et al., 2018). We generated *daf-7; dbl-1* double mutant and performed dauer assays (Fig. 7*A*). It was interesting to find that these animals also showed increased percentage in dauer formation compared with the control animals. Since DBL-1 and DAF-7/TGF-β are expressed and released from different cell types, there is a possibility that they initially act in parallel in a distributed neuronal network and maybe finally integrate the signals through their common Type II receptor DAF-4 (Estevez et al., 1993). We also performed chemotaxis experiments using AWC-dependent odors for *dbl-1* mutants. Our results showed that this double mutant combination of *daf-7; dbl-1* were also defective for AWC-sensed odors (Fig. 7*B*) as was the case for other double and triple mutants of *daf-7.* Data here support our above results that sensory cues from food are coupled to developmental dauer switch mediated through endocrine TGF-β pathways (DAF-7 and DBL-1).

## Discussion

Our study suggests that in *C. elegans*, chemosensory neural circuits are flexible and that their neuronal signaling is modified by developmental stages, their environment, the receptors expressed by the neurons and the signaling molecules secreted by them. Previous studies showed that EXP-1, an excitatory GABA receptor functions to modulate physiological behaviors of aggregation and bordering in the presence of food (Bendesky et al., 2012). N2 WT animals aggregate to avoid hyperoxia, population density, and stressful conditions such as aversive odors and food scarcity (de Bono et al., 2002; Chang et al., 2006). Food stimuli are important modulators of social behaviors as mutants prone to aggregation do not show behavior in the absence of food (de Bono et al., 2002). Chemosensory neurons (ASH, ADL, ASI) and their receptors (OCR-2, OSM-9, ODR-4, ODR-10) have been implicated in aggregation behavior (de Bono et al., 2002; Rogers et al., 2006). A study by Bendesky et al. (2012) has shown that social behaviors such as aggregation are dependent on many genes that show quantitative effects on behavior through genetic variations. This study also suggested that multiple receptors and ligand gated ion channels are suitable candidates for genetic variations with respect to their function in social behaviors in both *C. elegans* and humans (Bendesky et al., 2012). Same study indicated that EXP-1 may participate in detection of environmental signals (food) and hence modulate DAF-7 levels (Bendesky et al., 2012). Our initial experiments were based on the hypothesis that aggregation behavior is indirectly linked to sensory defects in food/attractive odors. Our results correlated with published results and our hypothesis where *exp-1* mutants were defective for AWC-dependent attractive odors and behaved normally toward odors sensed by other amphid neurons (AWA and AWB). In later experiments, our expression studies revealed that contrary to our expectation and its phenotypes, EXP-1 was not expressed in the AWC neurons but showed expression in the ASI neurons. The ASI neuron is also a chemosensory amphid neuron and modulates various behaviors in response to diverse environmental cues via DAF-7/TGF-β and other neurohormones released by it. Additional experiments showed that the chemotaxis defects were ASI neuron dependent and DAF-7/TGF-β was necessary for this defect.

The DAF-7/TGF-β pathway is a major regulator of dauer formation, an alternative growth stage. The dauer animals withdraw the ciliated endings from the amphid pore that is accompanied by changes in spatial organization of receptor proteins (SRD-1, STR-2, and STR-3; Lans and Jansen, 2006). Studies in the past have revealed that misexpression of proteins such as receptors and transcription factors can result in neuronal defects and altered functions (Sagasti et al., 1999). STR-2 is normally expressed in the AWC neuron but during adverse environmental conditions or in the absence of the DAF-7/TGF-β pathway it was strongly upregulated in the ASI neurons (Nolan et al., 2002). Direct correlation between ASI-specific expression of STR-2-dependent and dauer-dependent ASI cilia retraction was observed (Peckol et al., 2001). Neurons with retracted cilia can detect volatile odors (Ward et al., 1975; Bargmann et al., 1993; Peckol et al., 2001). Hence, STR-2 on expression in ASI neurons might be able to sense volatile odors. We also found that in case of *exp-1* mutants ASI ciliary endings were retracted and thus ASI might be able to sense volatile odors. However, *exp-1* mutants show defects toward all odors sensed by the AWC neuron, and there could be various reasons for this. First, it could be because of downstream synaptic connectivity of the sensory neuron. It is possible that ASI could elicit different responses to AWC-sensed attractants because AWC and ASI have different synaptic connections and release nonoverlapping molecules that could activate distinct circuits (White et al., 1986). Second, there could also be defect/s in the AWC neuron sensory ability because of changes at the ciliary receptive ends and the axon shape. In *exp-1* mutants, although AWC neuron cilia and axon shape was not obviously defective, it is possible that there are minor defects that we were unable to observe, that could alter the *C. elegans* response toward AWC-perceived odors. However, we found that loss of functional AWC neurons reproduced the *exp-1* chemotaxis defects. Thus, there might be a defect in AWC neurons in *exp-1* mutants which we could not detect. Third, we believe that animals with metabolic disorders could be defective toward sensory perception of food as has been shown previously (Fujiwara et al., 2002; Mak et al., 2006). Mutants in *exp-1* are defective in defecation and are thought to manifest a form of constipation (Beg and Jorgensen, 2003). Thus, it is also possible that because of defective metabolism they develop aversion to food related odors, and this could also be the reason for their increased aggregation and bordering on the sides of food during feeding.

Lowered expression of DAF-7/TGF-β is a significant marker of the dauer stage and *exp-1* mutants also decrease DAF-7 expression (Murakami et al., 2001). Here, we found that *exp-1* mutants were not dauer but *exp-1; daf-7* double mutant animals showed increased percentage of dauer animals similar to *daf-7* mutants. Thus, DAF-7 is epistatic to EXP-1 and may function downstream of EXP-1, an excitatory GABA receptor. Neuronal receptors/GPCRs are at the interface between the external milieu and the neuron. In a recent study, it was shown that STR-2 regulates life span in an ASI-dependent manner (Dixit et al., 2020), and it is well known that dauer animals can live for months as compared with reproductive animals that live for just a few weeks (Cassada and Russell, 1975). STR-2 expression levels are specifically increased in the ASI neuron during dauer like conditions (Lans and Jansen, 2006). This prompted us to investigate the physiological condition of the animal on removal of *str-2* from dauer forming animals such as *daf-7* mutants. Our data with these double mutants revealed that STR-2 is deployed in the ASI neuron to maintain the reproductive growth phase in the absence of *daf-7* or during other dauer inducing conditions such as food scarcity. Chemotaxis results indicate that EXP-1 functions upstream of DAF-7/TGF-β and EXP-1 also regulates DAF-7 levels (Bendesky et al., 2012). We reasoned that EXP-1 might also be contributing toward dauer formation. When the *exp-1* mutation was introduced in the *daf-7; str-2* mutant background, dauer formation increased to very high levels (∼80–100%) even at conducive growth temperatures of 16°C in the presence of food. Perhaps, both EXP-1 and STR-2 receptors are working in the ASI neurons to sense the environment (food) and contribute to the larval fate of the organism. The percentage of dauers increased with the loss of receptors (*exp-1; str-2*) as compared with *daf-7* mutants alone, indicating that the signals received here can also activate other signaling pathways in ASI. This is also supported by our rescue experiment where STR-2 expression in ASI neuron using extrachromosomal arrays brought down the percentage of dauers to WT levels since STR-2 overexpression might bypass *daf-7* loss and function through other signaling pathways to maintain normal growth. To address this idea, we analyzed another TGF-β protein DBL-1 and found that its deletion along with *daf-7* loss increased the dauer formation like *daf-7; str-2* and *exp-1; daf-7; str-2* mutants and showed chemosensory defects. Hence DBL-1 could be functioning in the absence of *daf-7* to maintain reproductive state along with EXP-1 and STR-2.

In this study, we have discovered that *exp-1* mutants are defective toward AWC-sensed odors. Furthermore, we have shown expression of EXP-1 in the ASI neuron which could explain the role of EXP-1 in DAF-7-dependent behaviors. We were able to identify new molecules participating in dauer formation and speculate that more genes/pathways are involved in the reproductive-dauer transition switch that could be detected in a sensitized background (summarized as model in Fig. 7*C*). Our study concludes that food derived attractive chemosensory cues can also be detected by ASI amphid neurons and this in turn could decide the developmental fate of the animal.

## Synthesis

Reviewing Editor: Erica Glasper, University of Maryland Decisions are customarily a result of the Reviewing Editor and the peer reviewers coming together and discussing their recommendations until a consensus is reached. When revisions are invited, a fact-based synthesis statement explaining their decision and outlining what is needed to prepare a revision will be listed below. The following reviewer(s) agreed to reveal their identity: Elizabeth Glater.

Overview

This manuscript explores EXP-1, a C. elegans excitatory GABA receptor, which has previously been reported to effect both bordering and aggregation behaviors, in the regulation of dauer formation and chemosensory behavior. The authors present novel data demonstrating that exp-1 mutants are defective in AWC-mediated chemotaxis. Experiments examine the expression pattern of exp-1 and observed that exp-1 is expressed in head neurons, including ASI (a chemosensory neuron whose expression of the TGF-beta neuropeptide daf-7 controls a wide array of C. elegans behaviors including dauer entry) and not expressed in AWC neurons. Expression of exp-1 in ASI neurons cell non-autonomously rescues the AWC-mediated chemotaxis defect. The authors further demonstrate that both daf-7 mutation and GPCR str-2, suppress the chemotaxis defect of exp-1 mutants, placing them downstream of exp-1 activity. While the mechanism underlying this epistasis is not explained in this manuscript, the cell non-autonomous action of exp-1 and its suppression by daf-7 are novel findings.

Although the manuscript presents many interesting findings, the manuscript requires a significant written revision. The manuscript 1) includes unnecessary background information that might be more appropriate for a comprehensive review, 2) contains many contradictory statements, and 3) sometimes includes logic that is difficult to follow. Detailed comments are below.

Major Comments

1. Many inconsistent statements plague this manuscript.

a. The title notes "dauer formation in a DAF-7 dependent manner", while the manuscript suggests exp-1 and str-2 regulate dauer formation in a daf-7 independent manner.

b. Seems to be a contradictory statement in the discussion (565-568). "Neurons with retracted cilia can detect volatile odors." Later in the manuscript it is stated that "In the case of exp-1 mutants ASI ciliary endings were retracted and thus ASI might not be able to sense volatile odors."

2. Significant questions were not answered.

a. Where is str-2 acting for the suppression of exp-1 chemotaxis defect? Given that the authors demonstrate that str-2 is not expressed in ASI neurons in an exp-1 mutant (Fig 4B), it then seems unlikely that str-2 would be acting there. The authors are encouraged to address this by rescuing exp-1. This can be done using the str-2 double mutant (Fig 4C) and bringing it back to exp-1 levels by expressing str-2 in either the ASI or AWC neurons under heterologous promoters.

b. The hypothesis that ASI neurons secrete a neuropeptide, DAF-7 or something else to aid in the cross-talk between ASI and AWC was not tested. Is it known whether AWC express DAF-7 receptors? The authors should find some way to manipulate signaling between ASI and AWC.

3. The authors should explain why they hypothesized that a relationship between aggregation behaviors and chemosensory behaviors exist.

4. The authors should provide information regarding whether dauers or non-dauers were tested in the chemotaxis assay. This information is present within the methods section; however, it should also be within the main text. Additionally, the implication of testing non-dauers should be included.

5. The authors should reference Zhang et al (DOI: 10.1074/jbc.M116.741132) as this paper demonstrates STR-2 is an olfactory receptor for 2-heptanone (an AWC-sensed odor).

6. The authors are encouraged to include a summary cartoon of the proposed model. While many unknowns may exist, this can simply be denoted by a question mark.

7. The authors reference a very important piece of work, that being the 2012 paper by Bendesky and colleagues. The findings of this paper should be explained in greater detail and all in one place. This will improve readability and highlight the importance of this paper to the field.

8. Greater attention should be given to identifying the strains and alleles used in this study. Authors should ensure that gene names are followed by the allele used in parenthesis. This includes within the text and within the graphs. This will increase the likelihood that experiments can be reproduced and results can be more interpreted. Should the authors prefer, this information could be presented in a strain table, but as currently presented, Table 1 is incomplete and does not include the necessary strain information.

Minor Comments

1. The authors are encouraged to represent statistical differences between groups using brackets with asterisks for all significant comparisons.

2. Please provide a reference for the following statement on Lines 136-137: "str-2 receptor normally expressed in the AWC neuron is upregulated in the ASI neuron during dauer stages..."

3. Data represented in Figure 7 is not discussed in the results section of the manuscript.

## Author Response

We thank the Reviewing Editor, Dr. Erica Glasper and the Reviewers’ for reviewing

our manuscript. The comments provided were insightful and have helped us improve

our manuscript and allowed us to present our results in a better and more convincing

manner. We have tried to address all the concerns of the Reviewers’ and are now

resubmitting a version of the manuscript that takes into account the Reviewers

concerns. We hope the Editor and Reviewers’ find that we have addressed their

questions and feel that our manuscript is now suitable for publication at eNeuro.

Major Comments

1. Many inconsistent statements plague this manuscript.

a. The title notes "dauer formation in a DAF-7 dependent manner", while the

manuscript suggests exp-1 and str-2 regulate dauer formation in a daf-7 independent

manner.

We have edited the manuscript carefully and have made efforts to address the

issues raised by the reviewers. We apologize about the mistake in the title and have

changed the title to "Dauer formation in C. elegans is modulated through AWC

and ASI dependent chemosensation"

b. Seems to be a contradictory statement in the discussion (565-568). "Neurons with

retracted cilia can detect volatile odors." Later in the manuscript it is stated that "In

the case of exp-1 mutants ASI ciliary endings were retracted and thus ASI might not

be able to sense volatile odors."

We have corrected this statement in the discussion (lines 570-572).

2. Significant questions were not answered.

a. Where is str-2 acting for the suppression of exp-1 chemotaxis defect? Given that

the authors demonstrate that str-2 is not expressed in ASI neurons in an exp-1

mutant (Fig 4B), it then seems unlikely that str-2 would be acting there. The authors

are encouraged to address this by rescuing exp-1. This can be done using the str-2

double mutant (Fig 4C) and bringing it back to exp-1 levels by expressing str-2 in

either the ASI or AWC neurons under heterologous promoters.

We thank the Reviewer for suggesting this good experiment. We performed the

experiment using both ASI (Pgpa-4) and AWC (Pstr-2) specific promoters expressing

STR-2 in the exp-1; str-2 double mutant. Chemotaxis assays were performed using

these transgenic lines and the results show that STR-2 likely functions in the AWC

neuron and not in the ASI neuron (extended Fig. 4 and lines 414-421).

b. The hypothesis that ASI neurons secrete a neuropeptide, DAF-7 or something

else to aid in the cross-talk between ASI and AWC was not tested. Is it known

whether AWC express DAF-7 receptors? The authors should find some way to

manipulate signaling between ASI and AWC.

2

Thank you for this suggestion. In order to address this concern we performed

imaging experiments to get more insight into DAF-7 receptor expression. Upon

performing imaging studies, we found that the DAF-7 receptor DAF-1 is expressed in

AWC neuron allowing us to hypothesize that DAF-7 secreted from ASI neuron could

be sensed by the AWC neuron through the DAF-1 receptors (Fig 3C and lines 356-

366).

3. The authors should explain why they hypothesized that a relationship between

aggregation behaviors and chemosensory behaviors exist.

We have explained the relationship between aggregation behaviors and

chemosensory behaviors in the discussion (lines 527-536)

4. The authors should provide information regarding whether dauers or non-dauers

were tested in the chemotaxis assay. This information is present within the methods

section; however, it should also be within the main text. Additionally, the implication

of testing non-dauers should be included.

Thank you for this comment. We have now added the required information to the

manuscript (lines 485-490)

5. The authors should reference Zhang et al (DOI: 10.1074/jbc.M116.741132) as this

paper demonstrates STR-2 is an olfactory receptor for 2-heptanone (an AWCsensed odor).

Thank you for this comment. We have referenced Zhang et al (line 411-414).

6. The authors are encouraged to include a summary cartoon of the proposed

model. While many unknowns may exist, this can simply be denoted by a question

mark.

Thank you for this suggestion. We have added a model figure to Fig. 7 (Fig. 7C) and

this has helped us better discuss our results.

7. The authors reference a very important piece of work, that being the 2012 paper

by Bendesky and colleagues. The findings of this paper should be explained in

greater detail and all in one place. This will improve readability and highlight the

importance of this paper to the field.

Thanks for the suggestion and we agree that it was an important piece of work

referenced for our study. We have now discussed their findings in the discussion

section (lines 537-544)

3

8. Greater attention should be given to identifying the strains and alleles used in this

study. Authors should ensure that gene names are followed by the allele used in

parenthesis. This includes within the text and within the graphs. This will increase the

likelihood that experiments can be reproduced and results can be more interpreted.

Should the authors prefer, this information could be presented in a strain table, but

as currently presented, Table 1 is incomplete and does not include the necessary

strain information.

We apologize that our strain table was incomplete and agree that allele information

is very important for others to repeat the experiments. We have now added the

genotypes and alleles for the strains to the table listing the strains used in this study.

Minor Comments

1. The authors are encouraged to represent statistical differences between groups

using brackets with asterisks for all significant comparisons.

We have added the required statistical differences in the figures as suggested and

hope that this will increase the clarity of the graphs.

2. Please provide a reference for the following statement on Lines 136-137: "str-2

receptor normally expressed in the AWC neuron is upregulated in the ASI neuron

during dauer stages..."

We have added the relevant reference and thank the Reviewer for indicating this

omission.

3. Data represented in Figure 7 is not discussed in the results section of the

manuscript.

We have now discussed the Fig 7 data in the separate results section as suggested

(lines 501-521).
